# A Hidden Pathway for Human Exposure to Micro- and Nanoplastics—The Mechanical Fragmentation of Plastic Products during Daily Use

**DOI:** 10.3390/toxics11090774

**Published:** 2023-09-12

**Authors:** Yang Yu, Nicholas Craig, Lei Su

**Affiliations:** 1 College of Marine Ecology and Environment, Shanghai Ocean University, Shanghai 201306, China; 2 School of Biosciences, The University of Melbourne, Parkville, VIC 3010, Australia; 3 Shanghai Engineering Research Center of River and Lake Biochain Construction and Resource Utilization, Shanghai 201702, China

**Keywords:** microplastic, pathways, mechanical fragmentation, exposure

## Abstract

In numerous environmental compartments around the world, the existence of micro- and nanoplastics (MNPs) in the environment has been verified. A growing number of studies have looked at the interaction between MNPs and human activities due to the risks they may pose to humans. Exposure pathways are key factors in measuring MNPs risks. However, current research largely ignores the contribution of mechanical fragmentation pathways to MNPs exposure during the daily use of plastic products. Our critical review demonstrated the research gap between MNP fragmentation and risk assessments via a network analysis. The release of fragmented MNPs and their properties were also described at various scales, with emphasis on environmental stressors and mechanical fragmentation. In the scenarios of daily use, plastic products such as food packaging and clothing provide acute pathways of MNPs exposure. The release tendency of those products (up to 10^2^ mg MNPs) are several orders of magnitude higher than MNPs abundances in natural compartments. Despite the limited evidence available, waste recycling, landfill and municipal activities represented long-term pathways for MNPs fragmentation and point sources of MNPs pollution in environmental media. Assessing the health effects of the fragmentation process, unfortunately, is further hampered by the current absence of human exposure impact assessments for secondary MNPs. We proposed that future studies should integrate aging evaluation into risk assessment frameworks and establish early warning signs of MNPs released from plastic products.

## 1. Introduction

As humanity enters the so-called plastic age, plastic products have pervaded almost every aspect of human life [1,2,3]. The toxicological impacts and ecological risks of the aforementioned compounds are being thoroughly assessed, particularly since microplastics and nanoplastics are of growing concern in environmental science and public health [4,5]. They have also been shown to have acute and long-term harmful effects in a range of models, including plankton and mammals, with adverse consequences ranging from individual mortality to behavioral abnormalities [6,7,8]. Whether micro- and nanoplastics (MNPs) at environmentally relevant conditions can likewise pose major dangers is, of course, still up for debate [9,10].

Humans are also inevitably exposed to MNPs in the environment via different pathways. Due to the early beginnings of plastic debris monitoring in marine organisms, a significant portion of which are considered as animal protein, there is a long history of research on the estimation of human exposure to MNPs via seafood consumption, typically of fish and mussels [11,12,13]. The risk of exposure via aquatic products is related to the specific part of the aquatic product consumed, e.g., MNPs contamination in the gastrointestinal tract and gills of fish does not fully reflect the risk of exposure [14].

In the field of public health research, the study of MNPs exposure pathways is more diverse, in that, in addition to the oral exposure pathways such as eating food and drinking water, a considerable number of studies have been conducted on atmospheric MNPs concentration levels and respiratory exposure pathways [15,16]. In terms of abundances, MNPs in the atmosphere can reach up to four orders of magnitude higher in indoor and urban environments, far exceeding the levels at which they have been detected in the same volume of aqueous sediments and organisms [17,18]. The great majority of these high levels of MNPs in the environment around humans are caused by the direct release of plastic products. This indicates that a significant amount of human exposure to MNPs may result from the regular usage of plastic products.

The sources of MNPs have usually been reported qualitatively or semi-quantitatively during the examination of exposure pathways in both indoor and field experiments. For instance, whereas bio-fragmentation during ingestion contributes to a minor portion of the MNPs in fish, in the calculations, they are all believed to originate from the environment [19]. Also, weathered plastic is frequently employed in exposure investigations to increase the environmental relevance of the data [20,21]; however, the secondary MNPs produced by these weathered plastics are rarely used for concentration corrections when calibrating concentrations. In fact, the majority of MNPs in the environment are secondary byproducts of the fragmentation of large items, with the exception of a small number of primary MNPs, such as pellets and micro-spheres [22,23]. Due to the limited production and use of manmade nanoplastics, the fragmentation process contributes almost the entire source of nanoplastics in the environment. Applying the theory of morphology, it has been shown that the surface structure of MNPs changes during fragmentation, for example, by increasing the specific surface area and complexity, that promote both the release of residual contaminants from the plastics and the enhancement of the level of adsorption of these secondary particles onto persistent organic pollutants (POPs) in the environment [24,25,26]. As a result, the secondary particles produced by the fragmentation of plastics have different physical and chemical properties, which raise the risk to the environment.

As the size of the MNPs observed continues to decrease, more and more focus is being placed on investigations of the fragmentation mechanisms, not the least of which is the application of polymer physics to explain them at the microscopic level [20,27]. Meanwhile, in the current body of toxicological research, the fragmentation of plastic items has rarely been considered as a potential exposure pathway for MNPs. Studies on various plastic products, including pacifiers, masks and drink bottles, have actually shown that a significant amount of MNPs is created by mechanical abrasion or by high temperatures and pressures, and that this quantity rises with usage [28,29,30]. These plastics come into close contact with humans during daily use until they enter the body via inhalation or food consumption. Unfortunately, the fragmentation of plastics that are directly related to human daily life has received little attention in current research, and toxicological studies using animal models have rarely taken into account the effect of fragmentation of aged plastics on concentration increases during exposure, even though this effect is critical in chronic and in situ experiments. The health risks of human exposure to aging plastics under conditions of daily use can only be completely addressed with a thorough understanding of the plastic fragmentation process and its implications for the production of secondary pollutants. To this end, based on an introduction to the toxicity and fragmentation mechanisms of MNPs, the current work will systematically review: (1) research trends related to plastic fragmentation mechanisms; (2) fragmentation scenarios and mechanisms of plastics products that are closely related to human beings; and (3) the significance of the fragmentation process in terms of the release of pollutants and their health risks.

## 2. The Research Trends and Knowledge Gaps

**Figure 1:**
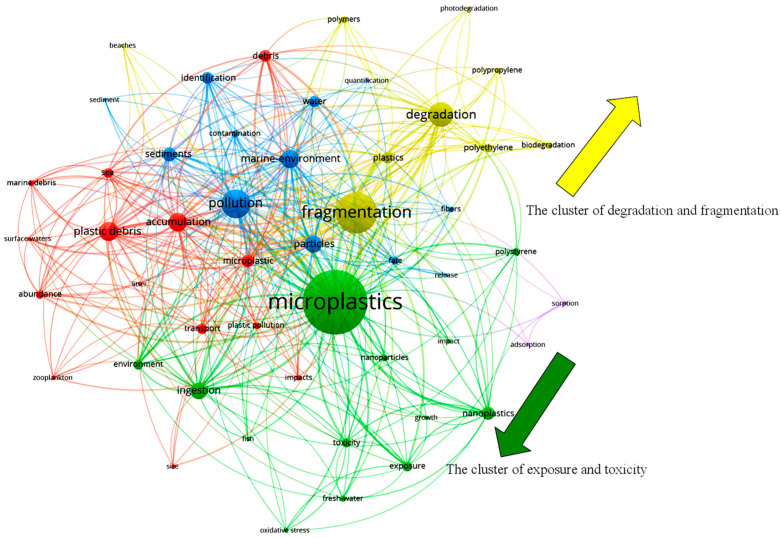
The clusters of keywords and connection analysis. The clusters are indicated by colors and the width of lines represent the degrees of connections.

In systematic reviews pertaining to pollutants, keyword-based bibliometric analyses have frequently been utilized to provide a visual depiction of research trends and hotspots in a particular research topic. We extracted all of the bibliometric information from 431 research papers using the keywords of fragmentation and microplastic. The search was conducted via WOS core selection on 15 June 2023. A network correlation was built based on the top 50 keywords from those papers (Figure 1). The research on fragmentation clearly focuses more on particular polymer types and degradation mechanisms around the main keywords. Instead, phrases associated with toxicity, exposure impacts, etc. and microplastics are grouped together under one category, and the other clusters also include research on marine litter and chemical adsorption. The aforementioned clustering patterns address the issue that only a small number of recent studies on plastic fragmentation have been taken into account when determining the risks of MNPs. The two core keywords, microplastics and fragmentation, are also split in research (Figure 1, direction of the arrow). Notably, the keywords related to chemisorption were independent of microplastics and fragmentation, suggesting that the environmental risk of MNPs releasing contaminants during fragmentation is missing in most of the toxicological studies. A viewpoint proposed that weathered plastic fulfils two of three criteria to impose a planetary boundary threat related to “chemical pollution and the release of novel entities” [31]. Similar ideas are echoed in some of the early studies of marine litter, that the aging process of plastics is an important contributor to the exacerbation of their ecological risks [23,32,33]. The current study trends make it evident that, in comparison with the aging mechanism, the risk of MNPs exposure derived from the fragmentation process is still not getting the attention it deserves. This could grossly underestimate the ecological risks of plastic debris in the field.

It is evident from the recent literature reviews that the conditions for fragmentation research are changing from ideal indoor settings to environmentally relevant scenarios. For example, Born et al. [34] concluded that, although fewer than half of all studies were conducted under environmentally relevant conditions, weathering and fragmentation experiments followed the transition from model to nature. Natural and daily-use conditions for plastic fragmentation are increasingly being incorporated into indoor accelerated or in situ aging simulations, and some studies have even gone so far as to directly use plastic fragments aged in the environment for the preparation of MNPs for exposure experiments [35,36,37]. Due to the lack of environmentally relevant conditions, however, a toxicity baseline of MNPs generated from fragmentation remains unclear. To this end, a full understanding of the roles of fragmentation is a fundamental task.

## 3. Stress-Induced Plastic Item Fragmentation Leading to the Production of MNPs

### 3.1. Environmental Stress

In fracture research, environmental stresses are frequently referred to as external forces that eventually induce material failure owing to corrosion brought on by solutions or other media [38]. From the perspective of MNPs, the major environmental stresses that can trigger plastic fragmentation are UV radiation, salt-water infiltration and temperature changes related to meteorological factors [39,40]. They are characterized by a long duration of effect, uniformity of action and diffuse distribution over the surface of the material. Historically, in order to test the performance and weathering resistance of plastic products, the roles of environmental stresses had been key external factors to be considered in the simulation of exposure. A number of in situ aging experiments have been set up directly in different latitudinal regions of the earth to explore the aging properties of plastics under long-term sustained environmental exposure [41]. In contrast to intuition, some thermoplastics can exhibit defects visible to the naked eye, such as cracks and surface delamination, after a relatively short exposure time (<1 month) [42]. These defects demonstrate the possibility of short-term environmental stress-induced formation of MNPs on the surfaces of plastic products.

**Figure 2:**
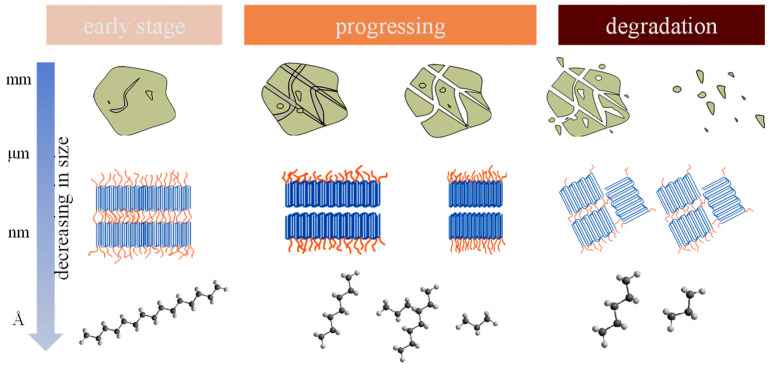
Observing the aging progress of polymers from the molecular (characterization using high-resolution SEM/TEM) to macro-scale (naked eye).

At various observational scales, polymers subjected to environmental stressors exhibit gradual changes (Figure 2). Within several years of UV exposure, polypropylene, for instance, will initially experience a decrease in the degree of polymerization or a reduction in chain length and molecular weight at the macromolecular level [27]. This modification will cause the polymer’s crystalline zone to grow, increasing crystallinity [43,44]. Additionally, it means that the materials would become more fragile and prone to breaking. Since the above-described aging process is irreversible under natural circumstances, different polymer materials’ propensity to release MNPs into the environment can potentially be anticipated using their thermodynamic characteristics. For instance, it is known via molecular simulations, e.g., crystallinity and hydrophobicity predictions, that polypropylene ages in artificial seawater more quickly than nylon, which means that polypropylene fishing gear will release MNPs earlier during its use [45]. Again, the formation of environmentally persistent free radicals on plastic surfaces was highly suspected as a trigger for degradation and physical damage [46]. It should be noted that projections based on the polymer properties of materials do not accurately reflect the true lifetime of plastics under complicated conditions of usage, much less the risks of exposure to microplastic emissions from the processes outlined above, due to the complexity of environmental pressures. As the most fundamental factor that causes plastic aging, the contribution of environmental stressors to the release of MNPs under actual-use conditions warrants further research.

### 3.2. Mechanical Abrasion and Wear

Mechanical abrasion and wearing damage plastics directly by actions including shearing, compression and tearing, in contrast to environmental stressors. In situ experiments have mostly concentrated on mechanical abrasion under the conditions of natural beach ecosystems, including the contributions of wave wash and sediment corrosion to fragmentation and MNPs generation [47,48,49]. The above studies have demonstrated that natural abrasion processes in the beach environment are a more important contributor to the nearshore accumulation of MNPs than the discharge of primary MNPs. It is clear that the mechanical abrasion resulting from the daily use of plastics is more intense and immediate than in natural conditions, but related studies in the area of MNPs have not been widely available until only very recently [34]. When we expand the scope of the study to the entire field of public health, the risk of inhalation of dust or particles represented by asbestos and synthetic leather has been systematically studied as early as the 1960s [50,51]. The acute causes of dust inhalation are essentially related to anthropogenic fragmentation, such as construction, metalworking and scrap recycling. They have shown mechanical fragmentation to be a significant contributor to the sources of hazardous compounds, despite the lack of a clear terminology.

**Figure 3:**
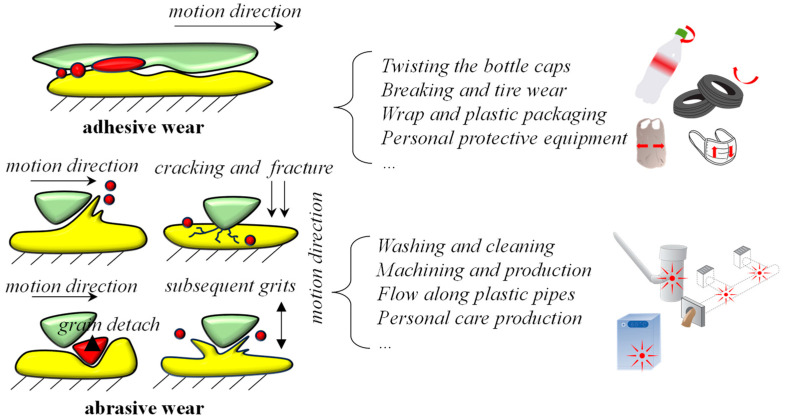
The generation of secondary MNPs in the processes of adhesive and abrasive wear and their representative activities.

The generation of MNPs via mechanical fragmentation is primarily derived from abrasive and adhesive wear, which are two of the most fundamental mechanisms involved in surface abrasion and wearing (Figure 3). For abrasive wearing, MNPs are formed when foreign particles collide with the surface of a plastic, and the direction in which these particles move has an impact on the shape of the MNPs. For instance, the vertical force of the particles can cause the plastic’s surface to crack, and, as the crack spreads, porcelain-tile-like secondary fragments are created. This could explain the cross-sectional microstructure and crack patterns of weathered plastic debris [52,53]. The particles tend to cleave into flaky secondary MNPs when they move at an angle or even parallel to the plastic’s surface. Such a phenomenon is obvious when inspecting the surfaces of micro-fibers collected from trail-running events [54]. Adhesive wear, in contrast, involves the contact and interaction of asperities on two surfaces with a strong adhesive force. Tire wear is thought to be one of the primary types of MNPs in road dust deposition due to adhesive wear such as braking [55,56,57]. In daily life, the opening and closing of plastic bottles represent typical adhesive wear and can form fatigue cracks after 10 min with twisting caps [58,59]. Plastic products that have been used or even aged are more susceptible to mechanical fragmentation than brand-new plastic products. Weathered plastic fragments, for instance, can produce more secondary MNPs during the shaking process than newly purchased ones [44,60]. This means that long-term environmental stressors operate as the basis for the majority of pathways for the generation of MNPs from mechanical fragmentation.

## 4. The Characteristics of Secondary MNPs Released by Fragmentation

### 4.1. Geometrical Features

**Figure 4:**
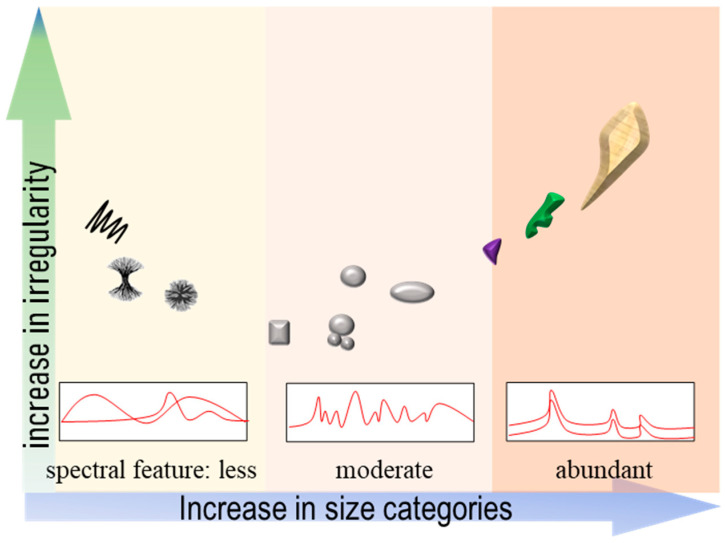
A diagram indicating how MNPs change when we “look” at them at different scales.

The geometrical features of MNPs largely varied by size category (Figure 4). For large particles, they were more likely to be generated via direct fragmentation. This resulted in irregular shapes being created such as sharp, asymmetric and coarse perforations. The structure of crystallization also played a role in shape formation. For example, weathered polyethylene tended to develop more strips than polypropylene due to its lamellar structure [44]. With decreasing size, the fragmented particle shape could be impacted from particle–particle and particle–wall collisions that are similar to fine sediment dynamics [61,62]. That would promote the generation of regular shapes with high degrees of roundness and sphericity. Recent field observations generally agreed with such deductions. In comparison with fiber, more fragments and spheres were found as part of the small microplastics and nanoplastics fraction [63,64]. The ultra-microstructure (1–10 nm) of nanoplastics is equivalent to the chain structures of given polymers to some extent. In this size category, there are substantial agglomerations of polymer chains that display a range of complex structures, e.g., lamellar and spherulitic. This is similar to the case of magnetite nano-crystals [65]. However, the geometrical features of ultra-small nanoplastics remain unclear due to the limited number of observations. The geometrical characteristics of MNPs are frequently associated with bodily harm. For instance, when considering broken plastic products, sharp cracks can scratch the skin and lead to infections. On a smaller scale, microfractures in MNPs may also damage the digestive tracts of organisms via respiratory and feeding pathways [7]. According to reports, smaller microplastics can penetrate cell membranes and cause an inflammatory reaction, which is a much more serious physical injury than that caused by larger plastics [66,67]. Unfortunately, little research has been conducted on the physical harm that MNPs cause to the human body as they fragment.

### 4.2. Surface Textures

Surface textures were too often ignored in MNPs characterization although they were closely related to the particle structure and chemical adsorption. If MNPs were directly generated by fractures and the breakdown of base materials, the surface textures denoted the variance of stress load and could be diagnosed using fractography. At this point, the surface textures exhibited certain patterns according to the polymer compositions and local modulus. Regarding nanoplastics in the field, they were primarily generated by embrittled and highly weathered surfaces and could show gradients of embrittlement deformation, e.g., brittle fractures and beach marks [27,53]. However, a smooth and flat surface was expected if particle erosion and collision continued in the weathering and aging processes. This mechanism was quite similar to the evolution of particle geometrical features as the surface patterns tended to be more regular. On the other hand, the surface texture roughly indicated the presence of insoluble material such as inorganic stuffing and additives. That meant that the surfaces of nanoplastics could be observed with embedded foreign matter and could be more distinct for small nanoplastics. Again, the ultra-microstructure of the surface texture is determined using chain structure crystallization. Further improvements in observation technologies are required. The surface texture of MNPs and the absorption of chemical contaminants are intimately connected. The vector effect of MNPs describes how a rougher surface has a higher specific surface area that allows the particles to adsorb more contaminants and deliver them to the body via different pathways [68,69]. The likelihood that POPs may enter the human body via MNPs can be readily identified if the surface textures of the individual particles are accurately quantified. On the other hand, MNPs with higher adsorption capacities may also be able to transport persistent pollutants from the organism’s gastrointestinal location to the outside of the body during digestion [68]. In order to prevent mistakes in the assessment of the risk of contaminant release, full partitioning and dissolved equilibrium concentrations should be taken into account when determining the actual risk of MNPs.

## 5. Human Exposure to MNPs via Mechanical Fragmentation

### 5.1. The Pathways of Acute Exposure

**Figure 5:**
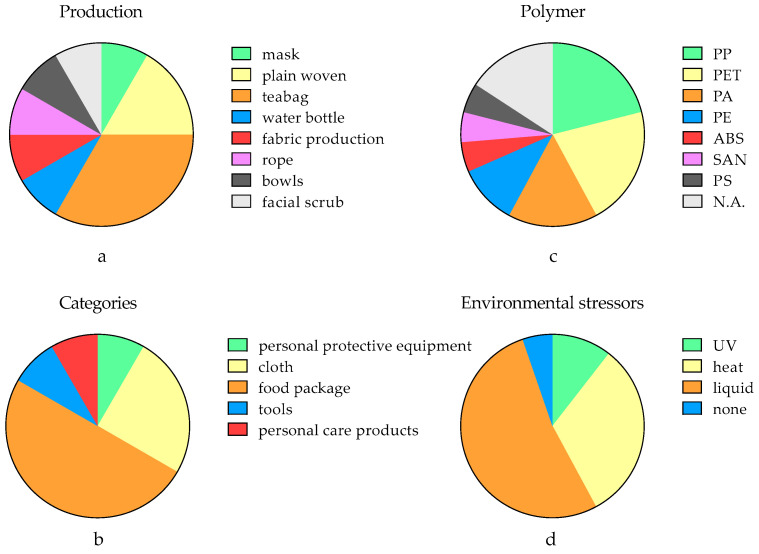
The distribution of the production types (**a**), categories according to the usage scenario (**b**), polymers (**c**) and environmental stressors (**d**) in the analyzed literature. The abbreviations: Polyamide (PA), Polyethylene (PE), Polyethylene Terephthalate (PET), acrylo-nitrile–butadiene–styrene (ABS), polypropylene (PP), melamine, polyethylene (PE), polystyrene (PS), and styrene–acrylonitrile (SAN); Not available in original references (N.A.).

The acute exposure pathways included the use of all kinds of everyday household items, such as cloth, personal protective equipment, food packaging and personal care products (Table 1). Due to its close relationship to human health and intensive use, more than 50% of the studies focused on food packaging such as teabags and water bottles (Figure 5a–c). This also results in a high level of environmental stress from liquid and heat (Figure 5d). Despite the difference in quantification, the levels of release tendency of those products (up to 10^2^ mg MNPs) are several orders of magnitude higher than MNP abundances in natural compartments, e.g., soil, water and biota [5,70,71]. The fragmentation process is more pronounced in the cases of beverage bottles and teabags because of their direct contact with liquids during usage and the potential for heating, which is primarily related to environmental pressures. In a study using controllable conditions, the use of hot water induced 10% more MNPs compared with the use of cold water [72]. Interestingly, not all environmental stressors significantly promote fragmentation. For example, the release of MNPs from rLDPE/LLDPE-modified asphalt during abrasion is quite stable at different water pH levels compared to other environmental factors [73]. Therefore, we must perform a complete screening for the major environmental stressors that contribute to fragmentation in the future. If we set 1000 nm as the benchmark for sub-microplastics and 100 nm for nanoplastics, only a limited number of studies have observed them in all MNPs (Table 1). This could be attributed to the low resolution of the detection techniques employed rather than the absence of smaller particles. Based on the surface fracture characteristics observed using SEM, friction marks can be produced on the surfaces of the particles even by means of washing, agitation, etc., suggesting the release of smaller-sized MNPs [30].

There are limited data on the impacts of human exposure to MNPs, despite studies being conducted on the fragmentation of plastic items in daily life. According to estimates based solely on paper cups, the average chronic daily intake was 0.03 mg MNPs/kg body weight per day [80]. This value is much higher than the quantity of microplastics that a person would normally consume by eating seafood [81]. In other words, it is preferable to check your apron for tears when preparing seafood than to be concerned about consuming an excessive amount of MNPs from your meal. Due to variations in measurements, such as the number of particles or the mass of the particles, which result in variances in the calculations, it is currently challenging to create a broadly applicable baseline of MNPs contamination from the fragmentation process. Alarmingly, recent simulations have demonstrated that low levels of shear force (4.0 kJ/L) are also capable of generating large quantities of MNPs, and that these smaller plastics can trigger apoptosis in fish cells [79]. In order to establish a baseline for chronic human exposure, it is necessary to determine the rate at which typical plastic products generate MNPs during usage using a standardized experimental setup.

### 5.2. Long-Term Exposure

In comparison with chronic and direct exposure, the long-term exposures from indirect pathways are more subtle, but their health risks to humans are significant. Based on the limited evidence, we confirmed that waste recycling, landfill and municipal activities represented pathways for the long-term exposure of humans to MNPs (Table 2). Waste recycling and landfill are the most typical forms of anthropogenic fragmentation of plastics by mechanical forces, as well as typical sources of pollutant emissions. In the first place, they are hazardous workplaces with a high abundance of MNPs, and onsite workers and nearby inhabitants are exposed to huge quantities of these particles, which are suspended during the fragmentation process and are inhaled via respiration. In addition to direct contact, wastewater, sludge and even drinking water are primary pathways for the transportation of secondary MNPs. The presence of MNPs in tap water has been confirmed by numerous studies and can be traced back to contaminated water sources, insufficient wastewater treatment and atmospheric deposition, which are thought to be the primary causes of tap-water pollution. There are only limited studies on the MNPs that are created when tap-water networks (e.g., kettles) break down [64,82,83]. As pipelines age, the water conveyance process may become a more visible source of MNPs contamination compared with the water source.

The usage of polymer composite materials is anticipated to enhance the danger of human exposure to microplastics in some areas where mechanical abrasion is substantial (e.g., highways and stadiums) [73,84,85]. Even with low-energy abrasion, MNPs can be released gradually. It was estimated that abrasive wear of shoe outsoles can produce up to 0.9 MNPs/linear meter/runner in trail-running events [54]. Long-term mechanical fragmentation mostly releases MNPs via adhesive wear and slow-acting compressive stresses. Therefore, the overall release of MNPs is significant compared with acute exposure pathways since these effects typically occur under conditions of significant environmental stress (e.g., fully open outdoor environments and fluid-filled pipes). The management of long-term exposure pathways will become more challenging as human development and outdoor activity increase together with the usage of novel materials. For environmental managers, reducing the long-term exposure pathways means not only controlling the sources of pollution but also monitoring and controlling the operation of various production and living activities.

**Table 2:** Potential pathways for long-term exposure of humans to MNPs.

Production	Pathways	Pollution Matrix	Environmental Stress	Primary Mechanical Abrasion	Release Tendency	Reference
water bottle	waste recycling	waste water/sludge	natural conditions	shearing force	967–24,798 MNPs/L; 773–1450 MNPs/kg	[86]
household plastic	waste recycling	waste water	natural conditions with UV	shearing force	110–200,000 MNPs/L	[87]
household plastic	landfill	surface soil	natural conditions with UV	compression	56–122 MNPs/5 cm^2^ samples	[88]
construction material	municipal activities	surface dust	liquid/heat	compression	0.53 ± 0.15 g MNPs/m^2^	[73]
plastic kettles	municipal activities	drinking water	liquid	compression	N.A. ^a^	[89]

^a^ Not available in original references (N.A.).

## 6. Outlook

### 6.1. Toxicological Approach Based on the Full Life Cycle of Plastic Products

Understanding the ecological risks of plastics is hampered by the compartmentalization of daughter productions, e.g., MNPs in aging and virgin plastic products in current studies. In indoor-exposure experiments, either brand-new polymer particles are used as exposure models or particles of already highly aged products are used as a substitute for real-world conditions. The aging of plastics, however, is a dynamic process and does not progress at a constant rate. The characterization and toxicological impacts of secondary pollutants, including MNPs and POPs, emitted at various stages of plastic aging must, therefore, be studied. This will make it possible to properly contextualize toxicological research using plastics and will serve as a foundation for future screenings of potential hazards of environmental contamination due to plastic waste on our planet.

### 6.2. Integration of Aging Evaluation into Risk Assessment Frameworks

For a long period, the evaluation of the aging degree was only used to assess the performance and service life of plastic products. There is a lack of consideration for these processes in the current risk assessment framework for MNPs, despite the strong relationship between the rate of aging, its byproducts and the health hazards connected to them. The health concerns that plastics may present to species, including humans, will be grossly understated by this omission. Therefore, aging degree should be taken into account as a significant indicator of forecast risk in future risk assessment frameworks. Additionally, quantitative methods should be employed to evaluate the contribution of mechanical abrasion and wear to the progression of aging in addition to studies of environmental stressors. The risk of human exposure to plastic products at various phases of usage and service life should be evaluated on this basis. We urge the investigation of new, less instrument-dependent imaging techniques or surface physics that may be utilized to rapidly determine the level of aging and contaminant release.

### 6.3. Establishing Early Warning Signals of MNPs Released from Plastic Items

The release of MNPs from plastic products is significantly variable depending on the polymer composition and processing techniques used. The release speed and the morphological properties of secondary MNPs are also influenced by variations in usage scenarios and the forms of mechanical fragmentation. Therefore, aging prediction models for common plastic items under various usage patterns, intensities and predicted lifetimes should be created in the future, and physicochemical markers that accurately indicate the progression of aging should be screened. Accelerated aging studies carried out indoors should be used to quantify the release rate of MNPs per unit of time or number of uses. We suggest that the fragmentation potential and environmental aging performance of various plastic materials be included in plastic risk assessment criteria together with the ongoing enhancement of the human health risk evaluation benchmarks for MNPs. This will be beneficial in assessing the hazards of long-term plastic exposure and can be used to direct infrastructure building, material choice and protective strategies for infrastructures that frequently come into contact with people.

## 7. Conclusions

Our current work has revealed hidden pathways regarding human exposure to MNPs via a literature review. The mechanical fragmentation of plastic products during daily use is a critical contributor to the secondary MNPs generated from abrasive and adhesive wearing. The physical features of those MNPs are determined by the size categories and mechanisms of fragmentation, which are closely related to their toxic effects and environmental behaviors. Acute and long-term pathways can be distinguished, with studies typically ignoring the latter. We proposed that future studies should use a toxicological approach based on the full life cycle of plastic products and include an aging evaluation in frameworks for risk assessment.

## Figures and Tables

**Table 1:** The acute exposure of humans to MNPs via multiple production means.

Production	Primary Mechanical Abrasion (Force Analysis)	Secondary MNPs	Release Tendency	Reference
mask	adhesive wear (compression and twisting)	fiber (0.5 mm to 3.8 mm)	24,300–55,900 MNPs/mask/d	[28]
plain woven	adhesive wear (compression and twisting)	fiber (0.1 mm to 1.5 mm)	51.6–107.7 mg MNPs/kg cloth	[74]
plain woven	none	fiber (0.1 mm to 1.5 mm)	20.6–59.9 mg MNPs/kg cloth	[74]
teabag	abrasive wear (particle collision)	fragments (N.A.)	0.9–1.1 mg MNPs/teabag/filter	[72]
teabag	abrasive wear (particle collision)	fragments (N.A.)	0.7–1.0 MNPs/teabag/filter	[72]
water bottle	adhesive wear (compression)	fragments (N.A.)	112–553 MNPs/L/cycle	[59]
teabag	abrasive wear (particle collision)	fragments (N.A.)	94% of samples released MNPs	[75]
teabag	abrasive wear (particle collision)	fiber (N.A.)	53.4–80.1 MNPs/kg teabag	[30]
fabric production	adhesive wear (compression and twisting)	fiber (N.A.)	mean at 114 mg MNPs/kg fabric	[76]
rope	adhesive wear (compression and twisting)	fragments (N.A.)	11–1052 μg MNPs/m rope	[77]
bowls	N.A.	N.A.	331–898 MNPs/treatment	[78]
facial scrub	abrasive wear (shear stress)	fragments (nano-sized)	up to 10^11^ MNPs/4g samples	[79]
